# CT-based 3D Super-resolution Radiomics for the Differential Diagnosis of Brucella *vs.* Tuberculous Spondylitis using Deep Learning

**DOI:** 10.2174/0115734056380084250720064859

**Published:** 2025-08-04

**Authors:** Kaifeng Wang, Lixia Qi, Jing Li, Meilan Zhang, Hai Du

**Affiliations:** 1 2nd Clinical Medical College, Fujian Medical University, Fuzhou, 350001, China; 2 Department of Radiology, Ordos Central Hospital, Ordos, 017000, China; 3 Graduate School, Baotou Medical College, Baotou, 017000, China

**Keywords:** Tuberculous Spondylitis, Brucella Spondylitis, Super-Resolution, Deep learning radiomics, CT imaging, Tuberculosis, Brucellosis

## Abstract

**Introduction:**

This study aims to improve the accuracy of distinguishing Tuberculous Spondylitis (TBS) from Brucella Spondylitis (BS) by developing radiomics models using Deep Learning and CT images enhanced with Super-Resolution (SR).

**Methods:**

A total of 94 patients diagnosed with BS or TBS were randomly divided into training (n=65) and validation (n=29) groups in a 7:3 ratio. In the training set, there were 40 BS and 25 TBS patients, with a mean age of 58.34 ± 12.53 years. In the validation set, there were 17 BS and 12 TBS patients, with a mean age of 58.48 ± 12.29 years. Standard CT images were enhanced using SR, improving spatial resolution and image quality. The lesion regions (ROIs) were manually segmented, and radiomics features were extracted. ResNet18 and ResNet34 were used for deep learning feature extraction and model training. Four multi-layer perceptron (MLP) models were developed: clinical, radiomics (Rad), deep learning (DL), and a combined model. Model performance was assessed using five-fold cross-validation, ROC, and decision curve analysis (DCA).

**Results:**

Statistical significance was assessed, with key clinical and imaging features showing significant differences between TBS and BS (*e.g.*, gender, p=0.0038; parrot beak appearance, p<0.001; dead bone, p<0.001; deformities of the spinal posterior process, p=0.0044; psoas abscess, p<0.001). The combined model outperformed others, achieving the highest AUC (0.952), with ResNet34 and SR-enhanced images further boosting performance. Sensitivity reached 0.909, and Specificity was 0.941. DCA confirmed clinical applicability.

**Discussion:**

The integration of SR-enhanced CT imaging and deep learning radiomics appears to improve diagnostic differentiation between BS and TBS. The combined model, especially when using ResNet34 and GAN-based super-resolution, demonstrated better predictive performance. High-resolution imaging may facilitate better lesion delineation and more robust feature extraction. Nevertheless, further validation with larger, multicenter cohorts is needed to confirm generalizability and reduce potential bias from retrospective design and imaging heterogeneity.

**Conclusion:**

This study suggests that integrating Deep Learning Radiomics with Super-Resolution may improve the differentiation between TBS and BS compared to standard CT imaging. However, prospective multi-center studies are necessary to validate its clinical applicability.

## 1 INTRODUCTION

Tuberculosis (TB), the ninth leading cause of global mortality, manifests severely as Tuberculous Spondylitis (TBS), affecting nearly half of TB patients and carrying a mortality rate of 8.6%. The increasing prevalence of TBS high-lights the critical need for early detection and treatment to miti-gate risks such as neurological complications and spinal defor-mity, emphasizing the importance of swift and accurate diag-nosis [1, 2]. Brucellosis, the leading zoonotic disease caused by *Brucella abortus*, also causes spondylitis in adults and poses risks of severe neurological and vascular complica-tions, underscoring the necessity of accurate early diagnosis [3, 4].

Both CT and MRI are capable of distinguishing BS from TBS, with CT being more effective in detecting bone changes [5]. However, differentiation remains challenging due to the heterogeneity of diseases that appear similar in imaging [6]. This situation points to an urgent need for an objective and precise method to evaluate images, which could improve differential diagnosis.

Deep learning-based radiomics may offer a solution. Artificial intelligence and its subtypes, particularly deep learning, are increasingly playing a significant role in all fields of medicine, such as in the diagnosis of colorectal cancer [7, 8], and the early classification of liver cancer lesions [9]. Research indicates that combining deep learning with conventional methods holds significant promise for clinical diagnosis and treatment [10, 11]. As a traditional deep learning model, ResNet is widely used due to its faster training, improved generalization, and robustness. Initially introduced by He *et al*. and trained on the ImageNet dataset, ResNet has proven effective in a variety of applications [12]. However, the efficacy of radiomics is often limited by image resolution, highlighting the need for high-quality images in model development [13].

Since the 1980s, Super-Resolution (SR) techniques have been introduced to enhance the spatial resolution of digital images by reconstructing higher-quality versions from lower-resolution observations. Generative Adversarial Networks (GANs) are often used for data enhancement along with image SR, through which the spatial resolution of the images can be improved [14]. Our research is dedicated to developing and comparing deep-learning radiomics models that utilize SR-CT for diagnosis and differentiation between TBS and BS.

## 2 MATERIALS AND METHODS

### 2.1 Patients

We confirm that this study was conducted in strict adherence to the principles of the Helsinki Declaration for research involving human subjects. This retrospective study was approved by the Institutional Review Board of Ordos Central Hospital (ID: 2020-007), with a waiver of written informed consent. The analysis covered the period from January 2018 to March 2023, during which 189 patients from Hospital 1 and Hospital 2 were evaluated based on predefined inclusion and exclusion criteria. Inclusion criteria included: (1) A confirmed diagnosis of Brucellosis Spondylitis or Tuberculous Spondylitis, (2) Absence of concomitant spinal pathologies (*e.g.*, degenerative spinal diseases, spinal tumors, or tumor-like lesions), and (3) Availability of complete clinical imaging data and CT examination. Exclusion criteria: (1) A history of vertebral surgery, (2) Spinal infections other than Brucellosis Spondylitis and Tuberculous Spondylitis (*e.g.*, pyogenic spondylitis) or co-infection of both, and (3) Poor image quality (*e.g.*, severe artifacts, motion artifacts, or low resolution affecting image analysis) [15]. Following thorough screening, 94 patients were included: 37 with Brucellosis Spondylitis (30 males, 7 females) and 57 with Tuberculous Spondylitis (28 males, 29 females). These patients were randomly allocated into a training set (n = 65) and a test set (n = 29) at a 7:3 ratio.

The clinical information of the Dataset and the CT image feature statistics are presented in Table **1**. The patient recruitment flowchart is demonstrated in Fig. (**1a**), and the overall study flowchart is depicted in Fig. (**1b**).

### 2.2 Acquisition of Clinical Baseline Characteristics and CT Images

For clinical data acquisition, age and gender were retrieved from the case management system. Regarding CT imaging, one center used a GE LightSpeed VCT and Discovery 750 HD CT scanner with tube voltages of 120 kVp or 140 kVp, where automatic tube current adjustments were made based on patient weight and size. Imaging parameters included a layer thickness of 5 mm with post-reconstruction at 1.25 mm. The second center also used a GE LightSpeed VCT, maintaining similar scanning parameters with tube voltages of 120 kVp or 140 kVp and tube currents between 200 to 300 mA, with a layer thickness of 5 mm, spacing of 5 mm, and post-reconstruction at 1.25 mm.

Two diagnostic CT specialists, with 8 and 10 years of experience, respectively, independently reviewed the images and evaluated key features such as the Parrot beak appearance, presence of dead bone, spinal deformities (kyphosis angle > 20°), psoas abscess, and severe intervertebral space stenosis (defined as intervertebral space height ≤ 1/2 of the average heights of the adjacent intervertebral spaces). Any discrepancies in data interpretation were resolved through discussion.

### 2.3 Image Preprocessing

To mitigate the multicentric effects in CT imaging, an image preprocessing procedure was performed using the OneKey platform [16]. The process included:

(1) Adjusting window width and window level (WW1000, WL250): This step optimized image contrast to standardize the image quality across different devices.

(2) Normalization to the range of 0-1: Min-Max normalization was applied to scale the pixel intensity values to a 0-1 range, eliminating intensity discrepancies [17].

(3) Resampling to 1x1x1 mm (nearest neighbor interpolation): The images were resampled to a uniform spatial resolution of 1x1x1 mm, preserving the original pixel values, ensuring spatial consistency and uniformity across different datasets [17].

### 2.4 3D Super-resolution

**Fig. (1):**
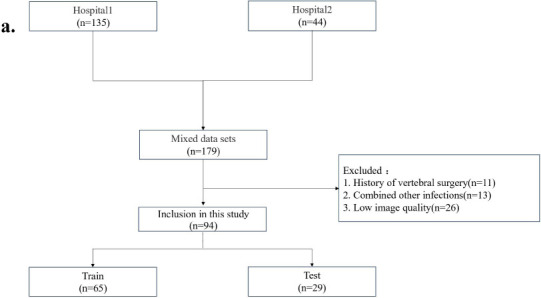
Study overview and imaging techniques. ( **a** ) Flowchart depicting patient recruitment and study design. ( **b** ) Workflow diagram outlining the radiomics analysis process used in this study, covering ROI Segmentation, Feature extraction, Feature selection, model construction and evaluation. ( **c** ) Standard CT image of the vertebra. ( **d** ) Super-resolution (SR)-enhanced CT image of the same vertebra (SR ×2), demonstrating improved clarity, texture, and edge definition. ( **e** ) Example of three-dimensional segmentation of a vertebra.

After preprocessing the original CT images, we employed a three-dimensional super-resolution technique based on Generative Adversarial Networks (GANs) to enhance the spatial resolution from 1 × 1 × 1 mm to 0.5 × 0.5 × 1 mm. This super-resolution pipeline was implemented using the OneKey platform [10, 16], which provides a standardized framework for deep learning–based medical image enhancement (https://github.com/OnekeyAI-Platform/onekey). This appro-ach utilized GAN as the core architecture, comprising a generator and a discriminator trained in opposition. The generator is designed to produce high-resolution images from low-resolution inputs, while the discriminator attempts to differentiate between real and generated images. Through this adversarial learning process, the generator progressively learns the underlying transformation between low- and high-resolution representations [18]. Further technical details of the GAN framework are presented in Supplementary File **1**. The original CT scans (Fig. **1c**) served as the visual reference, while the output images, referred to as SR-CT (Fig. **1d**), exhibited superior sharpness and enhanced visual detail.

### 2.5 Three-dimensional Segmentation and Radiomic Feature Extraction

Three-dimensional segmentation was performed using ITK-SNAP (version 3.8). Segmentation was performed by Radiologist 1 and Radiologist 2, with 5 or more years of experience, respectively. During the segmentation process, care was taken to avoid parts other than the cones. (Fig. **1e**) represents an example of three-dimensional segmentation. We used the Pyradiomics module for feature extraction (https://github.com/Radiomics/pyradiomics). Filters were used (Laplacian of Gaussian, LoG filter and Wavelet filter) to get more derived images and features.

The Interclass Correlation Coefficient (ICC) test was used to assess the inter-observer reliability of feature extraction. We randomly selected 30 CT images, Radiologist 1 and Radiologist 2, for ROI segmentation and feature extraction to assess the consistency of feature extraction. The consistency of feature extraction was better when the ICC was greater than 0.75.

### 2.6 Deep Learning Analysis

#### 2.6.1 Deep Learning Training

To determine the most suitable depth of the deep learning model for this task, the ResNet18 and ResNet34 models were selected [19]. We processed the largest Regions Of Interest (ROIs) into individual images, each corresponding to a specific patient, and resized them to 224 × 224 pixels. Training was performed with a batch size of 32 and an initial learning rate of 0.01. During deep learning training, the hyperparameters were kept consistent between ResNet18, ResNet34, and their corresponding SR models, including a batch size of 32 and an initial learning rate of 0.01. The specific settings are as follows:

To improve the model's generalization ability, a cosine annealing learning rate algorithm was used to adjust the learning rate dynamically. The formula (1) for the learning rate adjustment strategy is as follows:

**Table:** 

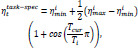	(1)

where, 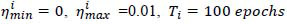 represent the minimum learning rate, maximum learning rate, and the total number of iteration epochs, respectively. Given that the backbone part uses pre-trained parameters, to ensure effective transfer learning, we fine-tuned the backbone parameters starting from 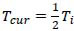.

Thus,the learning rate for the backbone is defined as in formula (2):

**Table:** 

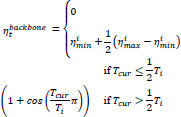	(2)

#### 2.6.2 Hyperparameter Configuration

- **Optimizer:** Adam, which combines the advantages of both AdaGrad and RMSProp optimizers.
- **Loss Function:** The Softmax Cross-Entropy Loss Function, suitable for multi-class classification tasks, was adopted. The Softmax function was used for the final fully connected layer, while other layers used ReLU (Rectified Linear Unit) as the activation function.
- **Dropout Usage:** Dropout was not used in the current model because the ResNet18 and 34 architectures we employed do not have Dropout layers by default.

#### 2.6.3 Deep Learning Feature Extraction

The AveragePooling layer was utilized in the deep learning models for feature extraction, resulting in a feature dimensionality of 512 for both the ResNet18 and ResNet34 models.

### 2.7 ComBat Compensation Procedure

The ComBat algorithm was used to reduce multi-center effects. It is based on an Empirical Baye (EB) framework, which enables it to effectively adjust for batch effects while preserving biologically or clinically relevant variability [20]. ComBat has demonstrated effectiveness in eliminating inter-center technical inconsistencies in radiomic feature values across PET, CT, and MRI data [21].

Before applying the ComBat method, all features were categorized into two groups based on hospital sources. The ComBat method was then implemented using the online tool available on the Sangerbox platform [22].

The specific procedure is as follows: Firstly, we merged the two datasets using the R package in SilicoMerging [23]. Furthermore, we used the method proposed by Johnson WE *et al*. to remove batch effects [20]. The final output was the processed dataset. As an example, we used ResNet34 and ResNet34-SR to generate density plots and Uniform Manifold Approximation and Projection (UMAP) visualizations of the dataset before and after ComBat for comparison of the effects. (Figs. **S1**, **S2**)

#### 2.7.1 Feature Fusion and Selection

For feature preprocessing, all features were normalized using Z-score normalization to ensure uniformity. Radiomics features with an Intraclass Correlation Coefficient (ICC) < 0.75 were excluded. Early fusion of deep learning features and radiomics features was performed, creating four distinct feature sets: clinical, radiomics, deep learning, and combined. Based on this, each dataset underwent the same feature selection process independently.

### 2.8 Feature Selection

Feature selection was performed to compare the differences between the two groups (BS and TBS), thereby identifying the optimal feature subset for each model.

#### 2.8.1 Clinical Feature Selection

The process began with the Mann-Whitney U test, retaining features with a p-value < 0.05. To reduce redundancy and enhance descriptive capacity, features with a Spearman rank correlation coefficient above 0.9 were limited to a single representative. A greedy recursive deletion strategy was then applied to further reduce redundancy. Finally, the Least Absolute Shrinkage and Selection Operator (LASSO) regression with 10-fold cross-validation was used for final feature selection, isolating relevant features for modeling.

### 2.9 Radiomics, DL, and Combined Feature Selection

The selection process for radiomics, DL, and combined features mirrored that of the clinical features. However, due to the high dimensionality of these features, the mRMR algorithm was applied before LASSO selection to retain the top 15 features [17], in order to prevent model overfitting. The specific steps included (1) Mann-Whitney U test, (2) Spearman rank correlation coefficient, (3) mRMR, and (4) LASSO.

The Minimal-Redundancy-Maximal-Relevance (mRMR) method is a powerful and widely used approach for feature selection in machine learning and data analysis. It combines two essential criteria for selecting an optimal subset of features from a high-dimensional dataset: maximizing relevance and minimizing redundancy [24]. A more detailed explanation of the Feature Selection algorithm is described in Supplementary File **2**.

### 2.10 Model Construction and Validation

Python's (3.90) scikit-learn (version: 0.18) was used for model construction and validation, creating four models: Clinical, Rad, DL, and Rad+DL, using a MultiLayer Perceptron (MLP) neural network [10]. Model training utilized a grid search algorithm, with 5-fold cross-validation, to determine the most suitable model hyperparameters. Model performance was evaluated by ROC curves and metrics like AUC and DCA. Additionally, results from the optimal model were used to build a nomogram to further enhance clinical utility.

### 2.11 Statistical Analysis

Group comparisons for categorical variables were conducted using the chi-square test or Fisher's exact test, whereas differences between groups for quantitative variables were assessed using the t-test or Mann-Whitney U test.

Statistical analyses were conducted at a significance level of 0.05 (two-tailed) using Python (version 3.9, http://www.python.org). A two-sided p-value < 0.05 was deemed statistically significant.

## 3 RESULTS

### 3.1 Baseline Characteristics of Patients

This study enrolled 94 patients (58 males, 36 females) with an average age of 58.38±12.39 years. Clinical and CT findings showed statistical significance for gender, parrot beak appearance, dead bone, spinal posterior deformities, and psoas abscess, with p-values<0.05 (Table **1**).

### 3.2 Development and Performance of Clinical Model

The clinical model's performance demonstrated an AUC of 0.936 for the training set and 0.726 for the test set (Table **2**).

### 3.3 Development and Performance of Radiomic Models

We extracted a total of 1,834 radiomics features and 512 deep-learning features. The radiomics features were categorized into seven types: (1) 14 shape-based features, (2) 360 first-order features, (3) 280 Gray-Level Dependence Matrix (GLDM) features, (4) 320 Gray-Level Size Zone Matrix (GLSZM) features, (5) 100 Neighboring Gray-Tone Difference Matrix (NGTDM) features, (6) 320 Gray-Level Run-Length Matrix (GLRLM) features, and (7) 440 Gray-Level Co-Occurrence Matrix (GLCM) features. Feature statistics are displayed in the Fig. (**S1a, b**). Radiomics features with an ICC > 0.75 were retained, resulting in 1,490 features for further analysis.

**Fig. (2):**
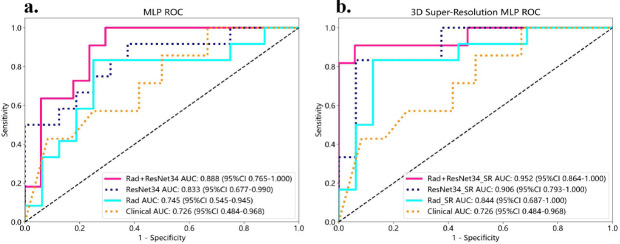
Model Performance Evaluation in the Test Cohort. (**a**) Receiver operating characteristic (ROC) curves for all models using a multilayer perceptron (MLP) algorithm on standard CT images. The receiver operating characteristic (ROC) curves for all models trained on standard CT images. The x-axis represents the specificity, and the y-axis represents the sensitivity. A larger area under the curve (AUC) indicates better classification performance. (**b**) ROC curves for all models using MLP on super-resolution CT (SR-CT) images.

In the test cohort, the AUC for Rad and Rad_SR were 0.745 (95% CI: 0.5446–0.9450) and 0.844 (95% CI: 0.6869–1.0000), respectively. ResNet34+Rad and ResNet34+Rad_SR AUC were 0.888 (95% CI 0.7650 - 1.0000), and 0.952 (95% CI 0.8642-1.0000), respectively. Further evaluation regarding the accuracy, sensitivity, specificity, and other models’ performance is shown in (Fig. **2**, Table **2**).

### 3.4 Development and Performance of DL Models

Two DL models, ResNet18 and ResNet34, were built for our study. Test set results showed improved model performance with SR, and ResNet34 demonstrated better performance. The AUC for ResNet18 improved from 0.797 (95% CI: 0.6300–0.9638) to 0.875 (95% CI: 0.7141–1.0000) with SR, and the AUC for ResNet34 improved from 0.833 (95% CI: 0.6769–0.9897) to 0.906 (95% CI: 0.7927–1.0000) with SR (Fig. **2**, Table **2**).

### 3.5 Development and Performance of Combined Models

We utilized the optimal DL model ResNet34 for further analysis, combined with the Radiomics (Rad) model, and used the combined model to integrate the outputs of the unimodal models.

The combined model demonstrated better performance compared to the clinical model and individual models, with ResNet34+Rad_SR showing improved performance. In the test cohort, the sensitivity and specificity of ResNet34+Rad_SR were high in this dataset (0.909 and 0.941, respectively), but further validation in larger, diverse cohorts is necessary.

A radar chart displaying the performance of different models on the test set is provided in Fig. (**S3**). According to the DCA curve, the ResNet34+Rad_SR model demonstrated a greater net benefit in distinguishing between TS and BS when the threshold probability exceeded 20% (Fig. **S3**). The model's calibration curve is shown in Fig. (**S4**).

### 3.6 Development of the Dynamic Nomogram

**Fig. (3):**
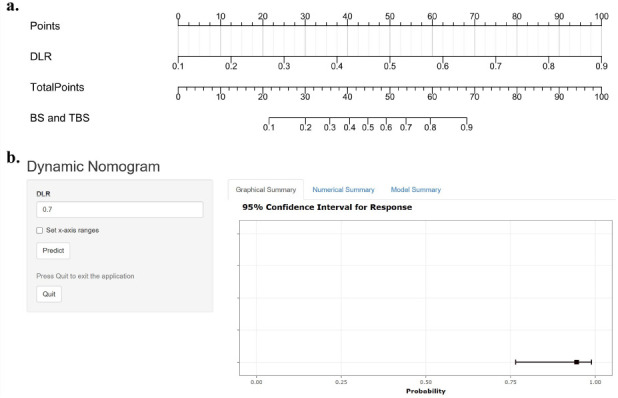
Nomogram and Online Calculator of the Optimal Model. (**a**) Nomogram for the Rad+ResNet34_SR Model – A graphical tool displaying the contribution of key predictive variables in the Rad+ResNet34_SR model. Each predictor is assigned a score, and the total score corresponds to a probability estimate for disease classification. (**b**) Online Calculator Interface – A web-based tool for individualized probability estimation. By inputting patient-specific parameters, clinicians can obtain real-time predictions based on the Rad+ResNet34_SR model.

The ResNet34+Rad_SR model was used to construct a no-mogram, and an online app was developed, accessible at the following: https://kaifeng-wang.shinyapps.io/dynnomapp-1/ (Fig. **3**).

## 4 DISCUSSION

Our study developed and validated deep learning radiomics models to differentiate TBS from BS using CT and enhanced SR-CT images. Comparative results consistently showed that the SR-CT model outperformed the CT model, indicating potential advantages in image quality and predictive accuracy.

### 4.1 Findings of this Study

Our clinical and CT feature analysis revealed significant results, with features like parrot beak appearance, dead bone, spinal deformities, psoas abscess, and gender showing a p-value under 0.05, indicating statistical significance. These findings are consistent with previous studies.

We created four models: Clinical, Rad, DL, and a combined model. The clinical model had the lowest performance. The Rad+ResNet34_SR model excelled, affirming the findings of Jun Zhang *et al*. on the superiority of the combined model [25]. This model, integrating 10 features with the greatest emphasis on DL features (n=7), suggested that quantitative insights derived from DL are pivotal for distinguishing BS from TBS. Among radiomics features, skewness, linked to bone density, was key in differentiating these conditions, stressing the diagnostic role of bone density [26]. Additionally, the application of wavelet transform, a method for detailed time-frequency analysis, was beneficial in highlighting lesion heterogeneity, enhancing the model's diagnostic accuracy [27].

Although the ResNet models used in this study offer potential advantages over other networks, with decreasing Top-1 and Top-5 error rates as network depth increases, this does not imply that blindly opting for deeper networks will yield better results. Instead, the optimal network depth should be determined based on the specific task and the available data volume [19, 28]. Therefore, we used ResNet18 and ResNet34 for a comparative study, finding that ResNet34 performed better. This aligns with findings from the study of Bingbing Xiao, who reported that ResNet34 outperformed both ResNet18 and ResNet50 in classifying microcalcification clusters. Xiao highlighted that shallow networks struggle to extract meaningful features, resulting in poorer performance compared to deeper CNNs, which are adept at identifying complex image features. However, Xiao also noted the risk of overfitting with excessively deep networks, underscoring the importance of balancing model depth to achieve optimal performance and generalizability [29]. It is worth mentioning that, in addition to selecting a ResNet model with an appropriate depth, this study employed a rigorous feature selection process to minimize the risk of overfitting, carefully controlling the optimal feature subset used to construct each model [30].

### 4.2 The Impact of Multi-center Imaging Variability and ComBat

In multi-center studies, the scanning devices, scanning protocols, and image reconstruction parameters used at different scanning centers vary, leading to significant variability in the imaging data [31]. This variability may introduce non-biological differences, reducing the model's generalizability [32]. Therefore, ensuring consistency in multi-center radiomics is crucial. ComBat, a post-reconstruction algorithm based on empirical Bayesian estimation [20], adjusts imaging features from different scanning centers to improve feature consistency. Studies have shown that ComBat correction can effectively eliminate inter-center technical inconsistencies in imaging feature values, enhancing the sensitivity of multi-center data research. It has recently been applied to multi-center PET, CT, and MRI data [21]. More recently, a new method called CovBat was proposed, which further reduces radiomic feature variability caused by different CT scanners compared to ComBat [33].

### 4.3 Comparison with other Studies

A study highlighted the remarkable capabilities of radiomics in distinguishing between BS and TBS, where the AUC for the comparison between BS and TBS (+) groups, utilizing a random forest combined model, reached 0.950. This achievement underscores the exceptional performance of radiomics in differentiating between TBS and BS [34]. However, compared to our study, the research did not incorporate DL features, focusing solely on radiomics attributes. Deep learning represents an emerging technique in image analysis, holding promising prospects for disease differentiation and diagnosis across various conditions [35, 36].

#### 4.3.1 3D Super-resolution

A major strength of our study is the application of a 3D super-resolution technique for medical images, which is based on the innovative architecture of Generative Adversarial Networks (GANs) [37]. This approach has been foundational in recent advancements in applying Deep Learning Super-Resolution (DL-SR) to radiomics analysis. Compared to existing radiomics studies, this AI-based (GANs) approach typically yields clearer images. This is largely due to GAN's ability to upscale low-resolution images to high-resolution ones, which may enhance model feature extraction and stability. In radiomics, where the foundational data comes from images, high-resolution images may inherently contain more potential and detailed information, as demonstrated by Hongqiang Xie *et al*., who enhanced CT image texture details for pneumonia using Self-Attention GANs (SAGAN) [38]. Additionally, considering the importance of accurate ROI delineation in radiomics studies—whether through manual or automatic segmentation—the clearer edge details provided by high-resolution images offer a potential advantage for precise ROI segmentation, particularly for tumors [39].

Image quality impacts the effectiveness of deep learning and radiomics models [14]. Therefore, the clear benefit of this technology is the improvement in the performance of existing models. As demonstrated in the study on Predicting the Prognosis of HIFU Ablation of Uterine Fibroids, the SR-DWI model outperformed the HR-DWI model across all machine learning algorithms [40]. Erick Costa de Farias *et al*. demonstrated the robustness of histology features from super-resolution, with 2X and 4X SR reconstructions showing excellent performance [13]. These studies highlight the ability of super-resolution to significantly improve medical image quality, thereby enhancing diagnostic accuracy and model robustness.

Artificial intelligence technologies, including deep learning, have been applied not only in the areas mentioned above but also in numerous Internet of Things (IoT) applications in recent years [41, 42]. Meanwhile, increasing attention has been drawn to the growth of the Internet of Things (IoT) in medical applications. In the last few years, technological developments in the surgical field have been rapid and are continuously evolving. One of the most revolutionizing breakthroughs was the introduction of the IoT concept within medical practice [43]. Beyond surgical treatments, IoT has also shown significant potential in areas such as infectious disease prevention and control, particularly in non-contact methods where it leads the way. Beyond surgical treatments, IoT has also shown significant potential in areas such as infectious disease prevention and control, particularly in non-contact methods where it leads the way [44]. Specifically, through wearable devices and environmental sensors, IoT can monitor patients' vital signs, pathogen exposure, and enable real-time disease monitoring, telemedicine, and patient management [45, 46]. Furthermore, by combining big data and artificial intelligence (such as machine learning and deep learning), IoT can analyze health data to predict disease transmission trends, thus optimizing public health response strategies [44].

### 4.4 Clinical Applicability

The models developed and validated in this study show potential value in differentiating Tuberculous Spondylitis (TBS) and Brucellar Spondylitis (BS). Radiomics and super-resolution techniques may enhance the model's feature extraction capabilities from imaging data. This radiomics model can serve as an auxiliary tool for radiologists, providing additional quantitative information, which may increase diagnostic confidence and accuracy, especially for junior physicians [47]. Furthermore, the super-resolution technology used in this study generates higher-resolution images, addressing critical medical imaging challenges such as long scan times, motion-induced image degradation, and high costs, particularly in improving MRI efficiency during emergencies like severe injuries or strokes [37]. This advancement reduces patient discomfort and financial burden. Finally, an online nomogram was created, enhancing model transparency and interpretability. Compared to traditional static nomograms, it offers simplicity and speed [48].

### 4.5 Potential Biases

Potential biases due to patient selection and imaging protocol variability must be acknowledged. The patient selection criteria could introduce bias, limiting the generalizability of the findings. Since patients needed to have complete clinical and imaging data of acceptable quality for inclusion, those with poor-quality images or missing data were excluded. This selection bias may result in an overestimation of model performance in real-world scenarios. Additionally, imaging protocols across different medical institutions may cause variability in radiomic feature extraction and model performance. Factors such as CT scanner manufacturers, reconstruction algorithms, and scan parameters (*e.g.*, tube voltage of 120 kVp *vs*. 140 kVp) could introduce inconsistencies. Despite using standardization techniques such as ComBat and ICC, residual differences may still impact model robustness. Therefore, future research should focus on more adaptable imaging standardization methods to enhance model applicability in diverse clinical environments. Furthermore, incorporating external validation and using various imaging protocols from different centers would help mitigate bias and improve generalizability. In a study with multiple independent external validations, MRI machines with different magnetic field strengths were purposely used to test model generalizability and robustness, providing a new approach to multi-center research validation [10].

## 5 LIMITATIONS

(1) Hand-crafted radiomics features rely on the experience of radiologists, indicating that future studies could benefit from automated deep learning segmentation to minimize subjective bias [49].

(2) As a retrospective study, our research may be influenced by selection and information biases. Reliance on historical patient records and imaging data could result in missing or inconsistent information. Moreover, retrospective studies cannot control confounding factors, which limit causal inference. Prospective studies are needed to mitigate these biases.

(3) Although data from two centers were used, the limited sample size restricts comprehensive external validation. Variations in imaging protocols and patient populations across institutions may affect model performance. The lack of independent external datasets may limit generalizability. Future multi-center studies with larger sample sizes are necessary to validate the model’s robustness.

Therefore, future research should focus on prospective, multicenter, and validation studies with larger sample sizes to further assess the clinical benefits of the model.

## CONCLUSION

This study investigated whether the integration of super-resolution CT and deep learning-based radiomics could assist in differentiating tuberculous from brucellar spondylitis. Our findings suggest that, with enhanced image quality, such models may support more accurate diagnostic differentiation between the two conditions. However, the clinical applicability of this model requires further validation. Future studies should incorporate external validation and larger-scale prospective research to assess its generalizability and practical clinical benefits.

## Figures and Tables

**Table 1:** Clinical and CT features of patients with TBS and BS.

**Characteristic**	**Total**	**TBS**	**BS**	***p*-value**
Age	58.38±12.39	58.75±14.41	57.81±8.56	0.4571
Gender	-	-	-	0.0038
Female	36(38.30)	29(50.88)	7(18.92)	-
Male	58(61.70)	28(49.12)	30(81.08)	-
Parrot beak appearance	-	-	-	<0.001
Negative	41(43.62)	35(61.40)	6(16.22)	-
Positive	53(56.38)	22(38.60)	31(83.78)	-
Dead bone	-	-	-	<0.001
Negative	55(58.51)	23(40.35)	32(86.49)	-
Positive	39(41.49)	34(59.65)	5(13.51)	-
Deformities of the spinal posterior process	-	-	-	0.0044
Negative	77(81.91)	41(71.93)	36(97.30)	-
Positive	17(18.09)	16(28.07)	1(2.70)	-
Psoas abscess	-	-	-	<0.001
Negative	62(65.96)	29(50.88)	33(89.19)	-
Positive	32(34.04)	28(49.12)	4(10.81)	-
Severe stenosis of intervertebral space	-	-	-	0.7289
Negative	50(53.19)	29(50.88)	21(56.76)	-
Positive	44(46.81)	28(49.12)	16(43.24)	^1^

^1^BS: Brucella Spondylitis; TBS: Tuberculous Spondylitis.

**Table 2:** Comparison of predictive performances of the models (Clinical, Radiomics, DL and combined models).

**Model**	**Cohort**	**Accuracy**	**AUC**	**95% CI**	**Sensitivity**	**Specificity**	**PPV**	**NPV**
Clinical	train	0.840	0.936	0.8850 - 0.9868	0.967	0.773	0.725	0.971
	test	0.632	0.726	0.4840 - 0.9684	0.857	0.545	0.500	0.857
Rad	train	0.844	0.946	0.8970 - 0.9943	1.000	0.744	0.714	1.000
	test	0.786	0.745	0.5446 - 0.9450	0.833	0.750	0.714	0.857
Rad-SR	train	0.859	0.915	0.8457 - 0.9840	0.960	0.795	0.750	0.969
	test	0.857	0.844	0.6869 - 1.0000	0.833	0.875	0.833	0.875
ResNet18	train	0.922	0.965	0.9230 - 1.0000	0.920	0.923	0.885	0.947
	test	0.750	0.797	0.6300 - 0.9638	0.917	0.625	0.647	0.909
ResNet18-SR	train	0.922	0.957	0.9116 - 1.0000	0.920	0.923	0.885	0.947
	test	0.857	0.875	0.7141 - 1.0000	0.750	0.938	0.900	0.833
ResNet34	train	0.938	0.981	0.9565 - 1.0000	0.960	0.923	0.889	0.973
	test	0.750	0.833	0.6769 - 0.9897	0.917	0.625	0.647	0.909
ResNet34-SR	train	0.859	0.935	0.8804 - 0.9893	0.880	0.846	0.786	0.917
	test	0.893	0.906	0.7927 - 1.0000	0.833	0.938	0.909	0.882
Rad+ResNet34	train	0.906	0.965	0.9278 - 1.0000	0.962	0.868	0.833	0.971
	test	0.821	0.888	0.7650 - 1.0000	1.000	0.706	0.687	1.000
Rad+ResNet34-SR	train	0.891	0.962	0.9230 - 1.0000	0.923	0.868	0.828	0.943
	test	0.929	0.952	0.8642 - 1.0000	0.909	0.941	0.909	0.941

## Data Availability

The data and supportive information are available within the article.

## LIST OF ABBREVIATIONS

MLP: multi-layer perceptron
Rad: radiomics
DL: deep learning
TBS: Tuberculous Spondylitis
SR: Super-Resolution
BS: Brucella Spondylitis

## STANDARDS OF REPORTING

STROBE guidelines were followed.
