# Supplementary material for: CT-based 3D Super-resolution Radiomics for the Differential Diagnosis of Brucella vs. Tuberculous Spondylitis using Deep Learning
Source: Curr Med Imaging. 2025 Aug 4;21:e15734056380084. doi: 10.2174/0115734056380084250720064859 (PMC13227580; doi:10.2174/0115734056380084250720064859)
Supplement: Supplementary file 1 [file CMIM-21-E15734056380084_SD1.pdf]

1573-4056/25 *Send Orders for Reprints to reprints@benthamscience.net*

BENTHAM SCIENCE

CrossMark

Current Medical Imaging

Content list available at: https://benthamscience.com/journals/cmir

Supplementary Material

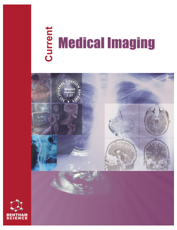

CT-Based 3D Super-Resolution Radiomics for the Differential Diagnosis of Brucella *vs.* Tuberculous Spondylitis using Deep Learning

Kaifeng Wang[1,#], Lixia Qi[2,#], Jing Li[2,3], Meilan Zhang[2,3] and Hai Du[2,*]

[1]2nd Clinical Medical College, Fujian Medical University, Fuzhou, 350001, China
[2]Department of Radiology, Ordos Central Hospital, Ordos, 017000, China
[3]Graduate School, Baotou Medical College, Baotou, 017000, China

Supplementary File 1

The three-dimensional super-resolution reconstruction approach in this study is based on a Generative Adversarial Network (GAN) architecture. GANs consist of two key components: a generator and a discriminator, trained in opposition to one another. Before model training, CT images are preprocessed to eliminate noise, correct artifacts, and normalize intensity distributions. High-resolution (HR) images are then downsampled to create corresponding low-resolution (LR) versions, forming LR-HR image pairs used for supervised training.

In the training process, the generator takes LR images as input and processes them through multiple convolutional layers, incorporating Batch Normalization and ReLU activations to extract visual features. The final layer of the generator applies a Tanh activation to produce synthetic high-resolution outputs.

The discriminator is tasked with distinguishing real HR images from those generated by the model. It also uses several convolutional layers, combined with LeakyReLU activations and Dropout layers to enhance generalizability and reduce overfitting. A Sigmoid activation in the final layer outputs a probability indicating whether an image is real or synthetic.

These two subnetworks are trained in a competitive setting: the generator aims to create visually convincing high-resolution images, while the discriminator improves its ability to detect fakes. Through this adversarial learning process, both networks iteratively refine their performance.

To optimize the generator's output, a compound loss function is applied, integrating three distinct components:

- Gradient loss, which promotes the retention of edge sharpness and structural detail by aligning the gradients of generated and real images.
- L1 loss, which quantifies pixel-level deviations between predicted and ground truth images using mean absolute error.
- Perceptual loss, which evaluates high-level feature discrepancies by comparing outputs from a pre-trained deep neural network, ensuring perceptual alignment with authentic HR images.

Together, these losses guide the network to generate super-resolved images with enhanced visual fidelity and structural realism, closely approximating true high-resolution CT scans.

More technical details are available at: https://github.com/OnekeyAI-Platform/onekey. The dataset used to train the 3D super-resolution reconstruction technique consists of millions of medical images.

DOI: 10.2174/0115734056380084250720064859, 2025, *21*, e15734056380084

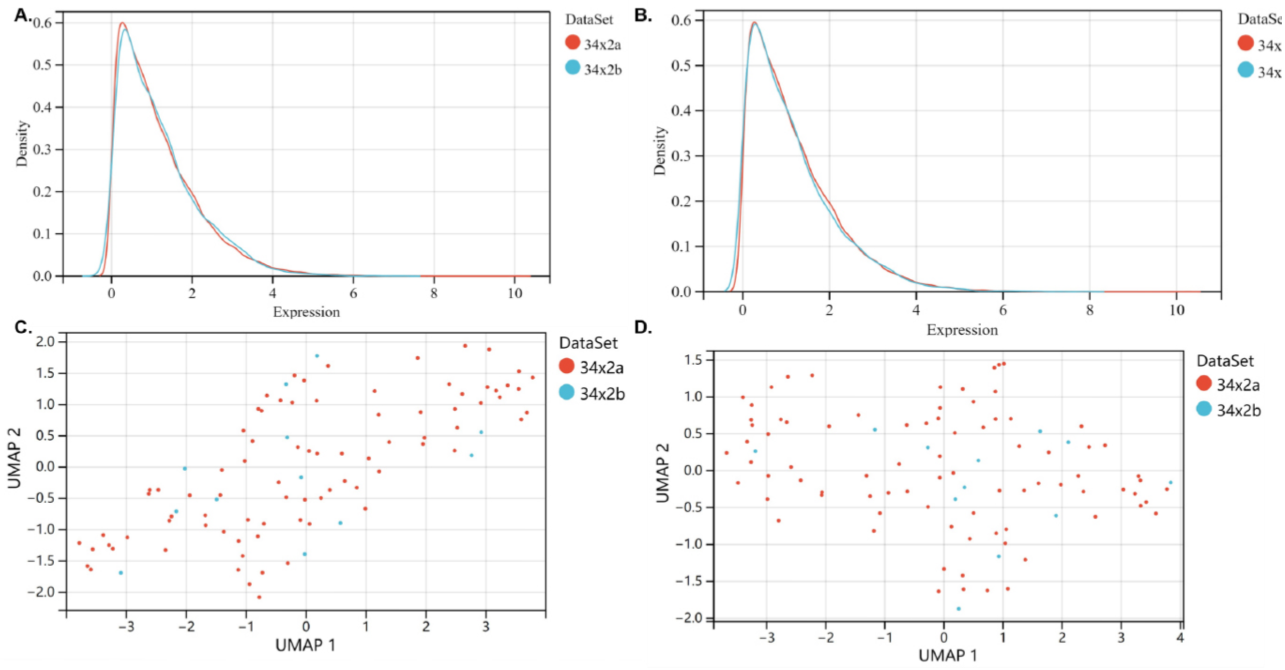

Fig. (S1). ComBat Algorithm for multi-center data harmonization.
(**a**) Density plot of the dataset before ComBat normalization, illustrating distribution differences between datasets caused by batch effects. (**b**) Density plot of the dataset after ComBat normalization, showing improved consistency in feature distribution across datasets. (**c**) UMAP visualization before ComBat normalization, highlighting the presence of batch-related clustering in the dataset. (**d**) UMAP visualization after ComBat normalization, demonstrating improved alignment and reduced batch-related variation, ensuring feature harmonization for model robustness.

Supplementary File 2

Mann-Whitney U Test:

This statistical method is primarily used to compare differences between two independent samples (BS and TBS in this study). In feature selection, the Mann-Whitney U test evaluates the distribution differences of each feature across different categories, identifying features with significant intergroup differences. Specifically, for each feature, the test determines whether its distribution differs significantly between the two groups. If the p-value is below a predefined threshold (0.05), the feature is considered discriminative.

Spearman Rank Correlation Coefficient:

This is a non-parametric measure of the monotonic relationship between two variables. In feature selection, it is used to detect correlations between features to reduce redundancy. When the Spearman correlation coefficient between two features exceeds a certain threshold (e.g., 0.9), it indicates a high correlation, suggesting that one feature may be redundant. In such cases, one feature is retained while the other is removed to mitigate multicollinearity while preserving descriptive capacity.

mRMR Method

The **Minimal-Redundancy-Maximal-Relevance (mRMR)** method is a powerful and widely-used approach for feature selection in machine learning and data analysis. It combines two essential criteria for selecting an optimal subset of features from a high-dimensional dataset: maximizing relevance and minimizing redundancy.

- **Maximal Relevance (MaxRel):** This criterion aims to select features that have the highest relevance to the target variable (class label). Relevance is typically measured using **mutual information** (MI), which quantifies the amount of information that one variable provides about another. The more mutual information a feature shares with the target variable, the more relevant it is. However, selecting features based solely on relevance can lead to the inclusion of redundant features that provide overlapping or similar information.
- **Minimal Redundancy (MinRed)**: To avoid redundancy, this criterion selects features that are as mutually exclusive as possible. Redundant features tend to convey the same information, which does not enhance the predictive power of the model but may instead lead to overfitting. The mRMR approach uses mutual information to evaluate and minimize redundancy among the selected features.

Least Absolute Shrinkage and Selection Operator (LASSO):

LASSO is a linear model that estimates sparse coefficients. This regression method, incorporating L1 regularization, performs feature selection while preventing model overfitting. By adding a penalty term based on the sum of absolute feature coefficients, LASSO forces some coefficients to shrink to zero, thereby achieving automatic feature selection. During the

selection process, LASSO identifies the most predictive features based on their linear relationship with the target variable while discarding less relevant or redundant features.

The combination of these methods effectively selects features that significantly contribute to model performance, reduces redundancy, and enhances model generalizability.

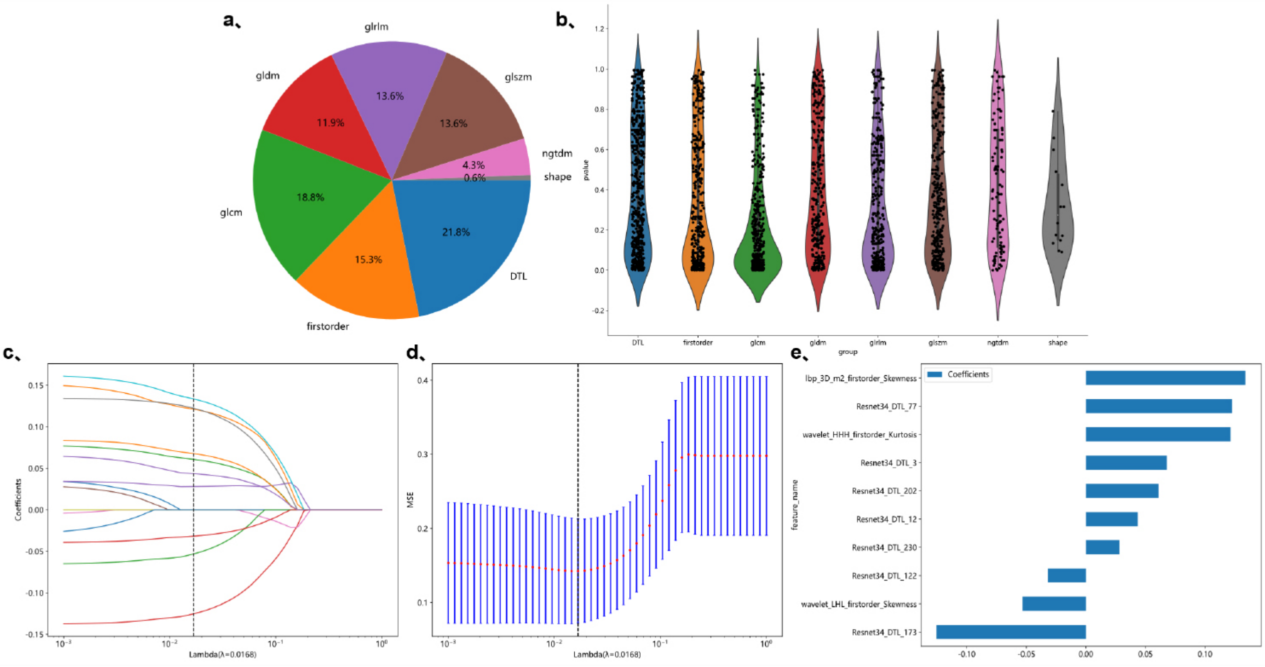

Fig. (S2). Radiomic and Deep Learning Feature Analysis (in model Rad+ResNet34_SR).
(**a**) Pie chart showing the distribution of radiomic and deep learning (DL) features, categorized by feature types.
(**b**) Violin plots illustrating the statistical distribution of radiomic and DL features. X-axis: Different feature categories (e.g., first-order statistical features, texture features, shape features, and deep learning features). Y-axis: p values of different feature categories.
(**c**) Coefficient paths of the Least Absolute Shrinkage and Selection Operator (LASSO) logistic regression model, validated across 10 folds, demonstrating the regularization process. X-axis: Log(λ), where λ is the regularization parameter.Y-axis: Regression coefficients of different features.
(**d**) Mean squared error (MSE) validation across 10 folds, indicating the optimal λ value selection for feature selection. X-axis: Log(λ), representing different values of the regularization parameter. Y-axis: Mean Squared Error (MSE) obtained from cross-validation.
(**e**) Feature weights of LASSO-selected nonzero coefficients displayed in a Rad-score histogram, showing the contribution of selected features.

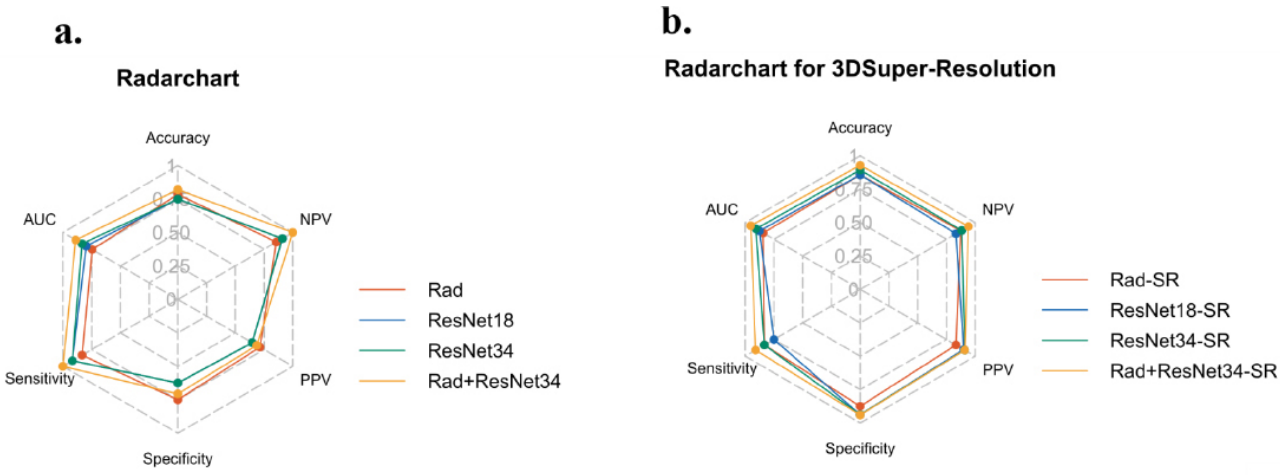

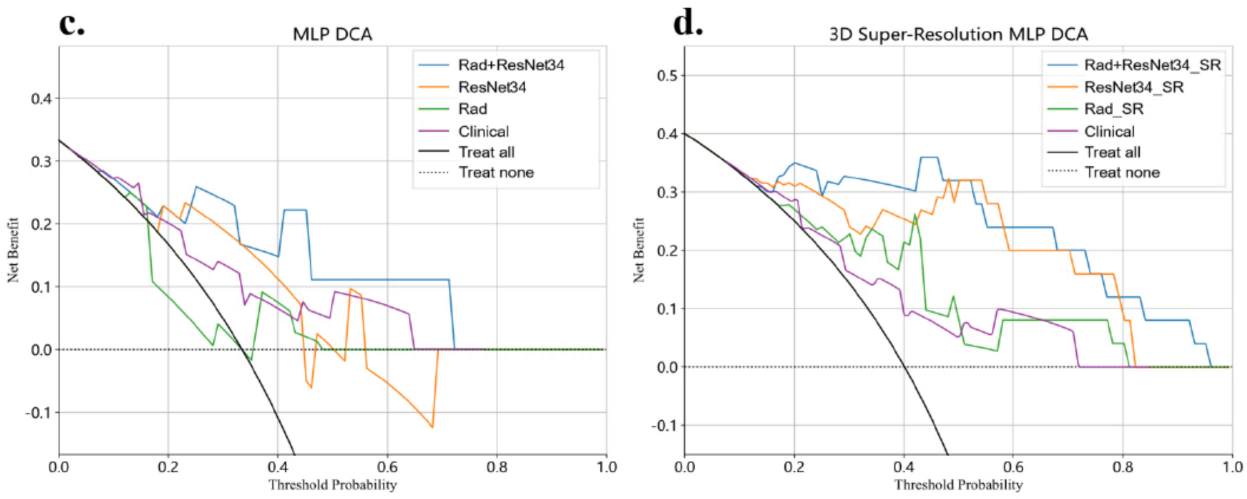

Fig. (S3). Model performance comparison.

(**a**) Radar chart comparing performance metrics (AUC, sensitivity, specificity, accuracy, PPV, and NPV) for Rad, ResNet18, ResNet34, and Rad+ResNet34 models.

(**b**) Radar chart comparing performance metrics for Rad-SR, ResNet18-SR, ResNet34-SR, and Rad+ResNet34-SR models, demonstrating the effect of 3D super-resolution enhancement on classification performance.

(**c**) Decision curve analysis (DCA) for Rad, ResNet18, ResNet34, and Rad+ResNet34 models, showing net clinical benefits across different threshold probabilities. X-axis: Threshold probability – The minimum probability at which a clinician would decide to treat a patient based on the model's prediction. Y-axis: Net benefit – A function of true positives and false positives, measuring clinical usefulness.

(**d**) DCA for Rad-SR, ResNet18-SR, ResNet34-SR, and Rad+ResNet34-SR models, evaluating the impact of 3D super-resolution enhancement on clinical decision-making. X-axis: Threshold probability, Y-axis: Net benefit

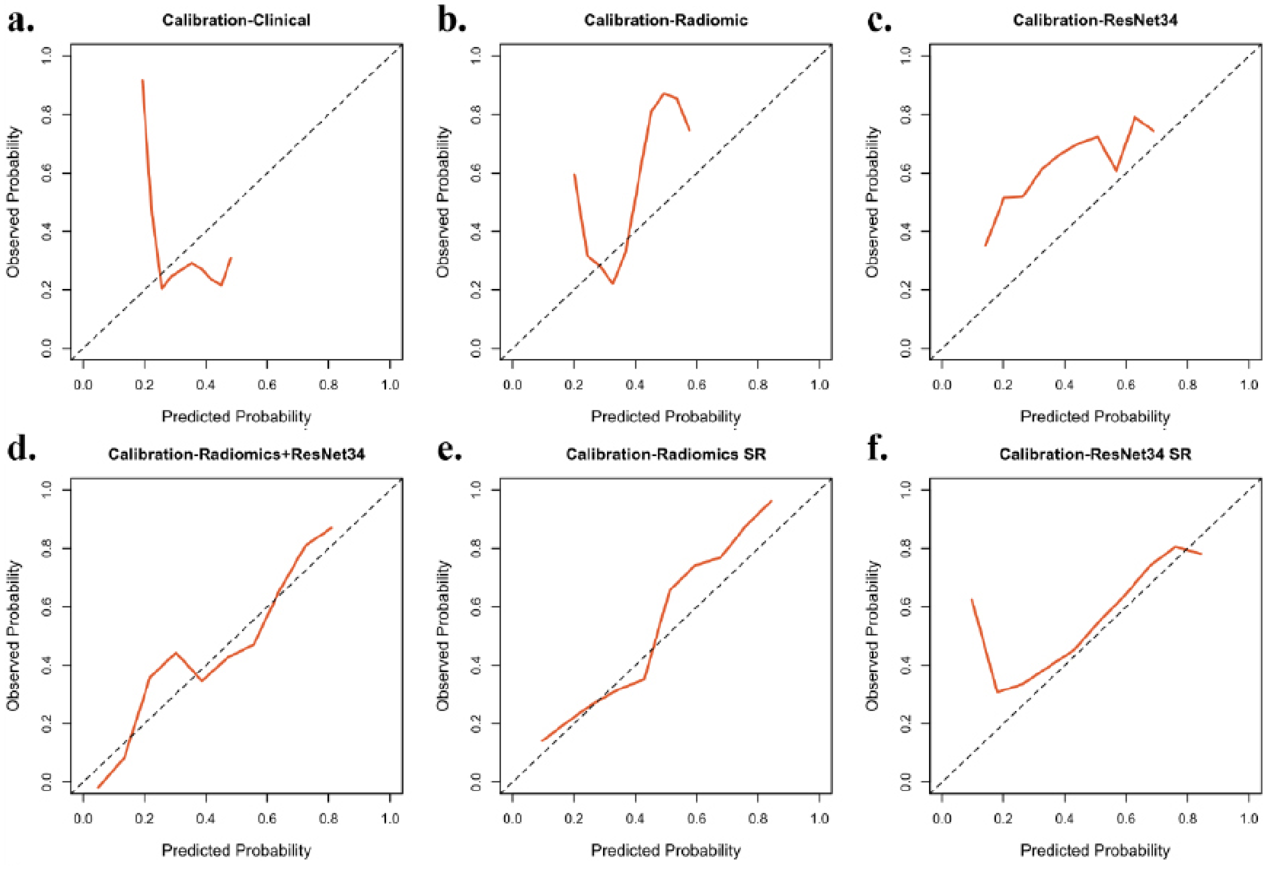

g.

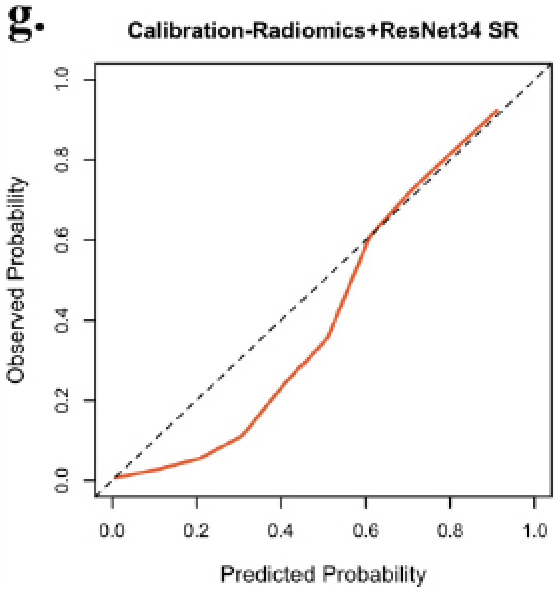

Fig. (S4). Calibration curves of different models.
Calibration curves evaluate how well the predicted probabilities from each model align with the actual observed outcomes. The diagonal dashed line represents perfect calibration, where predicted and observed probabilities are equal. Deviation from the diagonal indicates miscalibration (either overestimation or underestimation of risk).
Each subfigure represents a distinct model:
(a) Calibration curve for the Clinical model;
(b) Calibration curve for the Radiomic model;
(c) Calibration curve for the ResNet34 model;
(d) Calibration curve for the Combined Radiomics + ResNet34 model;
(e) Calibration curve for the Radiomics with Super-Resolution (Radiomics SR) model;
(f) Calibration curve for the ResNet34 with Super-Resolution (ResNet34 SR) model;
(g) Calibration curve for the Combined Radiomics + ResNet34 with Super-Resolution (Radiomics + ResNet34 SR) model

© 2025 The Author(s). Published by Bentham Science Publisher.

This is an open access article distributed under the terms of the Creative Commons Attribution 4.0 International Public License (CC-BY 4.0), a copy of which is available at: https://creativecommons.org/licenses/by/4.0/legalcode. This license permits unrestricted use, distribution, and reproduction in any medium, provided the original author and source are credited.
